# Bond-Reversibility Effects on Self-Crowding of Unimacromolecular Nano-Objects

**DOI:** 10.1021/acsmacrolett.5c00512

**Published:** 2025-09-11

**Authors:** Ainara Ruiz-Bardillo, Isabel Asenjo-Sanz, Ester Verde-Sesto, Lionel Porcar, Joachim Kohlbrecher, José A. Pomposo, Angel J. Moreno, Arantxa Arbe, Juan Colmenero

**Affiliations:** † Centro de Física de Materiales (CFM-MPC), CSIC-UPV/EHU, Paseo Manuel de Lardizabal 5, E-20018 San Sebastián, Spain; ‡ IKERBASQUE-Basque Foundation for Science, María Díaz de Haro 3, 48013 Bilbao, Spain; § 56053 Institut Laue-Langevin, 71 avenue des Martyrs, Grenoble Cedex 9, 38042, France; ∥ Laboratory for Neutron Scattering, 28498 Paul Scherrer Institut, CH-5232 Villigen, Switzerland; ⊥ Departamento de Polímeros y Materiales Avanzados: Física, Química y Tecnología (UPV/EHU), Apartado 1072, E-20080 San Sebastián, Spain; # Donostia International Physics Center (DIPC), Paseo Manuel de Lardizabal 4, 20018 San Sebastián, Spain

## Abstract

The scaling behavior of linear chains with reversible bonds and, in particular, its dependence on the concentration are fundamental problems of polymer physics that are not fully understood. By means of small-angle neutron scattering we investigate the conformations of reversibly bonding polymers from high dilution (where they form unimacromolecular nano-objects, usually known as single-chain nanoparticles) to crowded solutions and bulk state far above the overlap concentration (where they are expected to form a dynamic polymer network). Unlike the cases of simple linear chains with no bonds and of chains with strictly intramolecular irreversible bonds, no shrinkage is found, and the size and scaling exponent of the reversibly bonding polymers are essentially unperturbed by crowding. This is a relevant result that confirms the negligibility of many-body effects beyond the overlap concentration in crowded systems of reversibly bonding polymers and the validity of ultrasoft effective interactions for predicting their structural and phase behavior.

## Graphical abstract

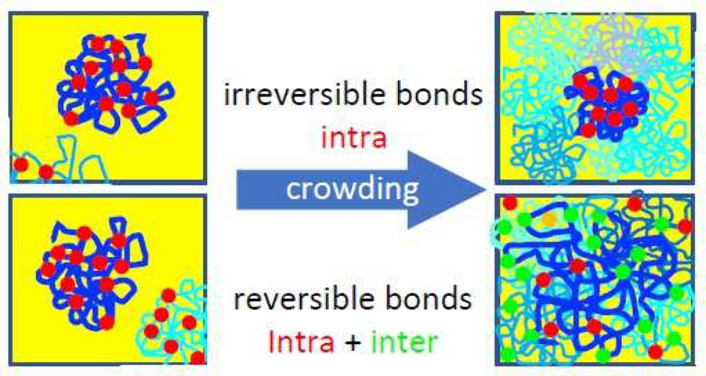

The scaling behavior of a linear polymer, characterized by the dependence of its size (e.g., radius of gyration *R*_g_) on its number of monomers *N* (*R*_g_ ∼ *N* ^ν^, where ν is the scaling exponent), is a fundamental problem in polymer physics. In good solvent conditions and high dilution, linear polymers adopt random self-avoiding walk statistics, where ν = ν_F_ = 0.589 is the Flory exponent.^1^ As the concentration increases, excluded volume interactions are screened, leading to ideal chain behavior (random walk statistics, ν = 1/2) in the bulk state. However, the crossover in the scaling properties over the entire concentration range from dilute to bulk conditions is not yet fully understood, particularly when intermolecular bonds (transient or permanent) are present. In the limit of high dilution, polymers with reactive sites form intramolecularly cross-linked objects known as single-chain nanoparticles (SCNPs).^2^ Because of their deformability that allows them to respond to external stimuli, in combination with the possibility of incorporating reactive sites or active species in their architecture, SCNPs have great potential in fields as catalysis, sensing, nanomedicine or as rheology modifiers to cite a few.^3–13^ The conformations of SCNPs at high dilution are usually sparse, with a scaling exponent of ν ≈ 0.5, and are dominated by the presence of short-range loops that are inefficient to fold the polymer into a compact state.^14^ However, at concentrated conditions and in the absence of intermolecular bonding (due to completion of intramolecular cross-linking at high dilution), irreversible SCNPs collapse into conformations of increasing compaction with concentration, resembling the fractal (“crumpled”) globular state (ν = ν_g_ ∼ 1/3) in bulk conditions. This result, initially found in simulations of dense solutions of irreversible SCNPs,^15^ has been tested experimentally up to now only in the case of irreversible SCNPs crowded by linear chains.^16–18^ The case of pure solutions of SCNPs (self-crowding) and their respective conformations still remains to be investigated in experiments.

If the bonds are reversible, the SCNPs are expected to lose their individual character as the concentration increases, and intramolecular bonds are exchanged with intermolecular ones. This leads to the formation of transient clusters and beyond the percolation threshold to a dynamic polymer network where reversible intra- and intermolecular bonds coexist. Recent simulations of a generic bead–spring model of reversibly bonding linear polymers have revealed unusual behavior of the scaling properties under clustering and network formation.^19^ The size and shape of the reversibly bonding chains exhibit a significantly weaker dependence on concentration compared to their nonbonding counterparts, and the scaling exponent is essentially unchanged. It has been suggested that the chains expand with respect to the nonbonded reference system, in order to increase exposure to the neighboring chains and to favor intermolecular bonding, which increases combinatorial entropy^20^ through clustering and network formation. A consequence of this result is that many-body interactions are averaged out to a flat energy landscape, and the two-body mean-force potential (effective potential between macromolecular centers-of-mass), which is derived at high dilution, is still able to describe with high accuracy the static correlations between the SCNPs’ centers-of-mass far beyond the overlap concentration.^21^ This finding is highly relevant since the description of the system as a fluid of ultrasoft particles interacting through the effective potential allows for the use of liquid state theory (e.g., integral equations or density-functional methods) and/or low-cost simulations to determine mesoscale organization and phase behavior. Ultrasoft potentials naturally emerge as effective interactions between macromolecular objects of high deformability (linear chains, stars, rings, etc.), and predict a rich variety of structural and thermodynamic behaviors in concentrated solutions.^22–24^ However, in general the emergence of the aforementioned many-body effects makes the approximation (and the most relevant predictions) progressively fail by increasing the concentration.^25,26^ The unusual possibility of using effective potentials to accurately predict structural and thermodynamic properties in concentrated solutions of reversible SCNPs, as anticipated in ref 21, is highly relevant, since these objects have the potential to be used as building blocks for designing smart materials that combine the characteristic functionality of SCNPs with the self-healing properties associated with the reversible bonds.^27–29^ More generally, there is an increasing interest in the design of advanced materials based on dual polymer networks or on interpenetrating networks, with two different responses to external stimuli associated with the two types of reversible bonds in the system.^30,31^ This design can be challenged by aspects as demixing of the two polymers^21^ or separation into a polymer-rich and a solvent-rich phase,^20^ which occur in regions of composition and temperature that can be anticipated by a low-cost calculation based on effective potentials.^21^

The main objective of the study presented in this article is to test in an experimental system the simulation findings, i.e., the very weak effect of concentration on the size and scaling properties of reversibly bonding polymers, by investigating the whole concentration range from reversible SCNPs at high dilution to dynamic networks far above the overlap concentration and in the bulk state. Moreover we also aim to observe the predicted collapse to fractal globular conformations in concentrated pure systems of irreversible SCNPs, a feature that up to now has been observed experimentally only when the crowders are linear chains. The only way to experimentally test these predictions is to apply scattering methods, where macromolecular conformations can be accessed through the scattering vector (*Q*) dependence of the measured intensities; specifically, small angle neutron scattering (SANS) techniques on properly isotopically labeled samples must be employed. In this work, we have exploited this unique opportunity of neutrons to demonstrate the decisive impact of bond-reversibility on the conformation of SCNPs in increasingly self-crowded environments.

Single-chain nanoparticles were obtained from linear copolymer precursor chains (Pre) of methyl methacrylate (MMA) and (2-acetoacetoxy)­ethyl methacrylate (AEMA; molar ratio 70/30; see Supporting Information, SI). Two molecular sizes were explored, corresponding to molecular weights *M*_w_ of 52 and 237 kg/mol. Irreversible SCNPs (MiNP) were synthesized by Michael addition^32^ leading to covalent cross-links, while Cu-complexation^14,33^ yielded reversible SCNPs (CuNP; see Scheme 1). To ensure formation of exclusively intramolecular cross-links leading to unimolecular nano-objects, the syntheses were carried out under high-dilution conditions (see SI). The success of the procedures is illustrated in Figure 1 for the low-molecular weight systems (investigated using the SANS-I instrument at the Paul Scherrer Institut), where SANS results in dilute solutions (5 mg/mL) are shown. In such dilute conditions, intermolecular interactions are negligible, the associated structure factor is close to unity and the scattered intensity *I*(*Q*) directly reflects the macromolecular form factor *P*(*Q*):^34^

1 $$I\left(Q\right)={\left(\rho_{p}-\rho_{s}\right)}^{2}nV_{p}^{2}P\left(Q\right)$$

**Figure 1:**
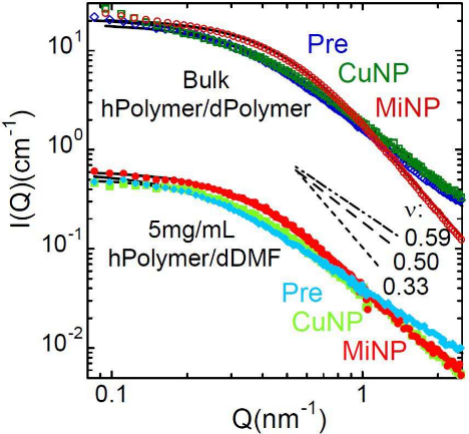
Neutron coherent intensity scattered by diluted solutions of protonated chains in deuterated DMF at 5 mg/mL (filled symbols) and bulk labeled samples (empty symbols) of low-molecular weight precursors (blue diamonds), MiNPs (red circles), and CuNPs (green squares). Lines are fits of generalized Gaussian coils. Straight lines indicate the slopes for the intensity in the fractal regime corresponding to values of ν = 0.59 (dashed-dotted), 0.50 (dashed), and 0.33 (dotted).

**Scheme 1:**
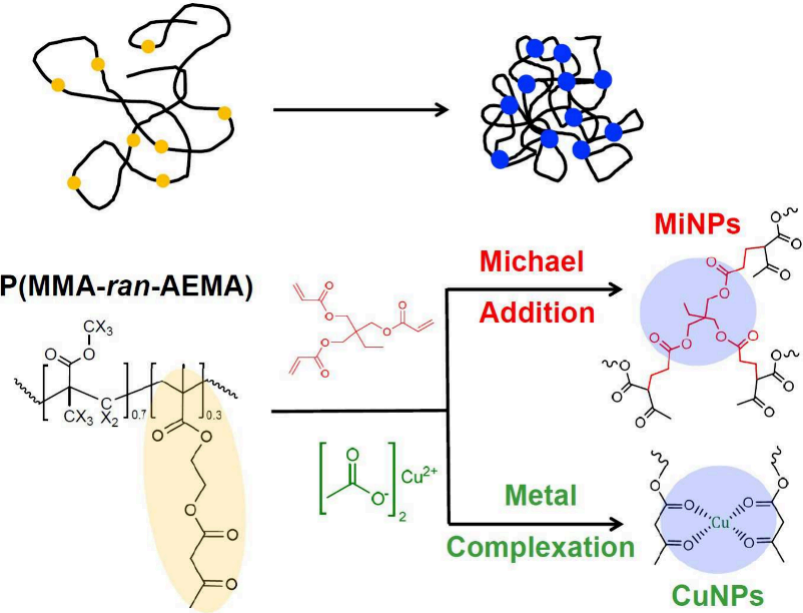
Upper Part: Schematic Representation of a Linear Precursor Chain and a SCNP Obtained through Internal Cross-Linking Processes^a^

a Yellow circles represent the reactive groups, and blue circles represent cross-links. The lower part shows in detail the two particular synthesis routes followed in this work.

Here, ρ_*x*_ is the scattering length density (SLD) of component *x* (*x*: polymer (p) or solvent (s)); *n* is the number of chains per unit volume, and *V*_p_ is the volume of a polymer chain. Due to the large difference in scattering length of protons and deuterons (*b*_H_ = −3.7406 fm vs *b*_D_ = 6.671 fm), the scattered intensity can be enormously enhanced if one of the components is protonated and the other one is deuterated. Therefore, the SANS experiments on the solutions shown in Figure 1 were carried out using deuterated dimethylformamide (dDMF) as solvent and protonated chains. The chain form factor *P*(*Q*) containing information on intramolecular correlations reflects the chain dimensions, quantified via the average radius of gyration ${\mathrm{R̅}}_{g}$ , as well as the degree of compaction of the macromolecules, revealed by the above introduced scaling exponent ν. The overall size determines the *Q*-range where *P*(*Q*) starts to decay. For larger macromolecules, this happens at smaller *Q*-values. The scaling exponent ν dictates the slope at intermediate *Q*-values (fractal regime), where *P*(*Q*) ∝ *Q* ^–1/ν^.^1^ As mentioned above, higher ν-values correspond to more swollen chains. In the two limiting cases of compaction for a macromolecule, namely, the self-avoiding walk conformation and the globular morphology, ν = ν_F_ = 0.59 and ν = ν_g_ = 1/3, respectively and for the intermediate case of a random walk, representing the Gaussian conformation of a linear chain in a θ-solvent or in bulk, ν = 1/2. Simple inspection of the solution results in Figure 1 reveals that the chain dimensions are reduced and the macromolecule is more compact after intramolecular cross-linking. From a quantitative analysis of *P*(*Q*) in terms of generalized Gaussian coil functions^35^ (see SI) the values of ${\mathrm{R̅}}_{g}$ and ν are obtained. The initial precursor value of ${\mathrm{R̅}}_{g}$ = 6.4 nm is reduced to ${\mathrm{R̅}}_{g}$ = 4.9 and 4.5 nm for CuNP and MiNP, respectively, concomitant with the decrease of the ν-value from that expected for linear chains in good solvent 0.59 to 0.48 and 0.42 in CuNP and MiNP. Obtained reversible SCNPs are sparser than irreversible SCNPs, a usual observation that can be rationalized using Flory arguments and assuming that actually only the nonbonded segments contribute effectively to the excluded volume and entropic terms of the free energy.^14^ It is noteworthy the SANS results for the SCNPs do not show significant signatures of aggregation, ensuring that the macromolecules are isolated nano-objects and the bonds are purely intramolecular in dilute solution. From the ${\mathrm{R̅}}_{g}$ values, overlap concentrations $c^{*}=M_{w}/\left({\left(2{\mathrm{R̅}}_{g}\right)}^{3}N_{A}\right)$ (*N*_A_: Avogadro’s number) of about 41, 92, and 120 mg/mL are deduced for Pre, CuNP, and MiNP, respectively.

Determining the form factor of individual macromolecules within a concentrated solution is not trivial since, under crowded conditions, the interferences between different macromolecules are also reflected in the scattered intensity. However, the different interaction of neutrons with protons and deuterons allows for this by applying high-concentration labeling methods.^34^ In these experiments, solutions of mixtures of identical macromolecules, one species protonated and the other deuterated, are investigated. Defining ϕ as the volume fraction of the deuterated chains in the solute, the coherently scattered neutron intensity becomes

2 $$I\left(Q\right)=\phi \left(1-\phi \right){\left(\rho_{p,H}-\rho_{p,D}\right)}^{2}nV_{p}^{2}P\left(Q\right)+{\left[\phi \rho_{p,D}+\left(1-\phi \right)\rho_{p,H}-\rho_{s}\right]}^{2}\times nV_{p}^{2}S_{t}\left(Q\right)$$

As in eq 1, *n* is the total number of macromolecules per unit volume, and now ρ_p,H_(ρ_p,D_) refers to the SLD of the protonated (deuterated) chains. Thus, the scattered intensity contains two terms: one related with the single chain form factor of the macromolecules *P*(*Q*), and thereby reflecting intramolecular correlations, and another with the total structure factor, *S*_t_(*Q*), that embodies information on the total (both intra- and intermolecular) correlations between monomers. Isolating intramolecular correlations is possible if the prefactor of *S*_t_(*Q*) equals zero:

3 $$\phi \rho_{p,D}+\left(1-\phi \right)\rho_{p,H}-\rho_{s}=0$$

This is the zero-average contrast (ZAC) condition, where the average SLD of the polymer molecules (summed over the deuterated and protonated species) matches the SLD of the solvent. This condition can be satisfied by tuning the value of ϕ for a given solvent, or looking for the solvent with the proper SLD for a chosen ϕ-value. It is advantageous to follow the second option and fix ϕ ≡ 0.5, which is the value that maximizes the contribution of the scattered intensity from intramolecular origin in which we are interested (first contribution in the rhs of eq 2). Then, eq 3 reduces to

4 $$\rho_{p,D}-\rho_{s}=\rho_{s}-\rho_{p,H}$$

To apply this strategy, deuterated macromolecular counterparts were synthesized (see SI). SANS experiments on solutions of either protonated or deuterated chains with different solvent compositions were performed to find this matching condition, which turns out to be satisfied with a deuterated solvent volume fraction of about 0.44 (see SI).

The results of the experiments on the low-molecular weight samples under ZAC conditions for different total polymer concentrations below and above c* are shown in Figure 2. For Pre and MiNP samples of solutions with concentrations up to 400 mg/mL could be prepared. Highly concentrated solutions of CuNPs are extremely difficult to handle, they become gel-like, and therefore, the solution with maximum concentration explored in this case was 200 mg/mL. Experiments on the limiting case of bulk samples (no solvent) were also conducted and are also included in Figure 2. The behavior of reversible SCNPs is qualitatively different from that of precursors and irreversible SCNPs. The form factor of CuNP shows very little effect upon crowding, while Pre and MiNP present clear compaction signatures when the polymer concentration increases. The differences between the bulk conformations of the three macromolecules can be appreciated in Figure 1 (results are represented by empty symbols). Michael irreversible SCNPs’ dimensions and scaling exponent are again clearly smaller than those of precursor chains, while reversible CuNPs show rather similar conformation to that of the precursors.

**Figure 2:**
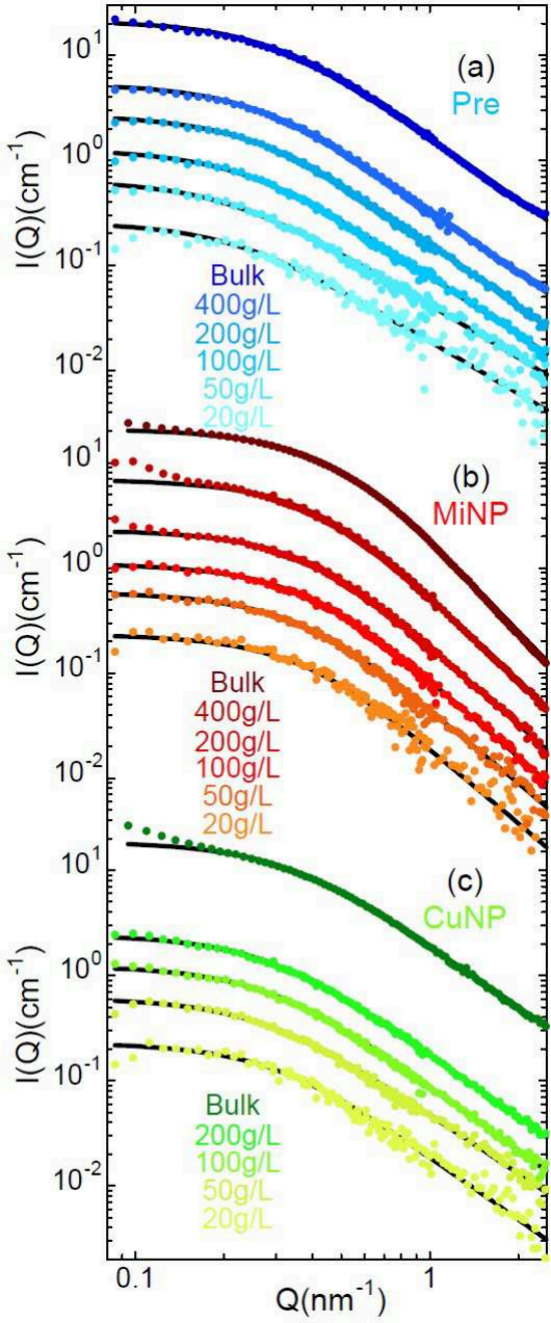
Neutron coherent intensity scattered by solutions of low-molecular weight chains in ZAC conditions for precursors (a), irreversible SCNPs (MiNP) (b) and reversible SCNPs (CuNP) (c) for the total polymer concentrations indicated and in bulk. Lines are fits of generalized Gaussian coils.

The values of the parameters of the generalized Gaussian coil $\left({\mathrm{R̅}}_{g},\nu \right)$ describing the SANS results on the low-molecular weight samples are displayed in Figure 3. In the vicinity of c*, the radius of gyration of precursor and irreversible SCNPs starts to decrease with respect to the value in the dilute regime, while the dimensions of the reversible SCNPs remain practically unperturbed over the whole concentration range investigated. Their scaling exponent is also constant, within the uncertainties. The MiNP form factor is described by a ν-value of 0.42 in dilute conditions, and ν starts to decrease around c* until reaching a value close to 1/3 in the bulk. As expected, precursors’ results show the extended and Gaussian conformations at high dilution and in bulk, respectively, also crossing over in the neighborhood of c*. We also note that the good agreement with the results from high dilution in full contrast condition (results in Figure 1 and also on deuterated SCNPs in protonated DMF, see Figure 3) supports the consistency of the experimental procedure and the good matching conditions.

**Figure 3:**
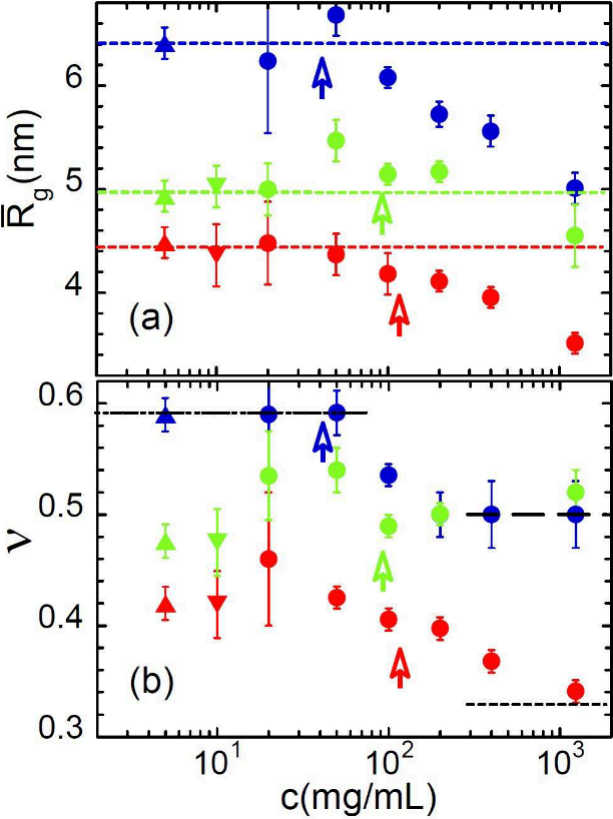
Parameters characterizing the macromolecular conformation of low-molecular weight precursor chains (blue symbols), MiNPs (red symbols) and CuNPs (green symbols) as a function of polymer concentration: (a) average radius of gyration ${\mathrm{R̅}}_{g}$ ; (b) scaling exponent ν. Results correspond to full-contrast (protonated polymer in deuterated solvent: up-triangles, inverse label: down-triangles) and ZAC condition (circles). Vertical arrows indicate the value of c* in each case. Dotted lines in (a) show the ${\mathrm{R̅}}_{g}$ -value in dilute conditions and lines in (b) the values of ν = 0.59 (dashed-dotted), 0.50 (dashed), and 0.33 (dotted).

Analogous experiments were performed employing the D22 instrument at the Institut Laue Langevin on the high-molecular weight samples.^36,37^ In this case, the overlap concentration is lower, and thus, the explored solution concentrations correspond to stronger crowding conditions. Moreover, the fractal regime extends down to lower *Q*-values, which allows for a more accurate determination of the scaling exponents. Figure 4 shows the results corresponding to ZAC conditions for polymer concentrations of 20 mg/mL (close to c* for these samples) and 200 mg/mL. The differences in the slopes of the intensity in crowded conditions are highlighted in these bigger macromolecules, nicely showing that the behavior is very similar to that commented on above for the smaller chains in bulk conditions (Figure 1). In Figure 5 ${\mathrm{R̅}}_{g}$ and ν values obtained from fits are represented as a function of the ratio between the concentration and c*, corroborating the behavior of the low-molecular weight samples displayed in Figure 3 and providing further experimental support to the predictions of the simulations.

**Figure 4:**
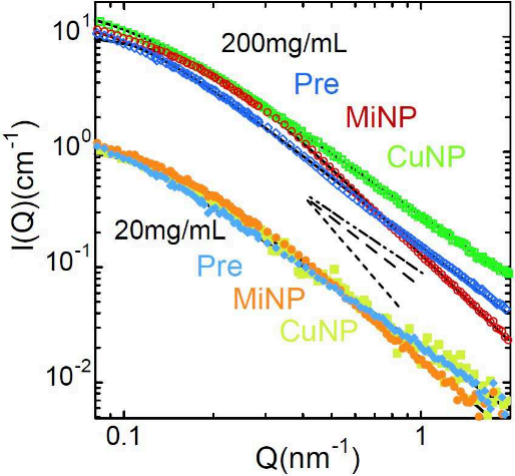
Neutron coherent intensity scattered by solutions of high-molecular weight chain in ZAC condition at 20 mg/mL (filled symbols) and 200 mg/mL (empty symbols) for precursors (blue diamonds), MiNPs (red circles) and CuNPs (green squares). Solid lines are fits of generalized Gaussian coils. Straight lines indicate the slopes for the intensity in the fractal regime corresponding to values of ν = 0.59 (dashed-dotted), 0.50 (dashed), and 0.33 (dotted).

**Figure 5:**
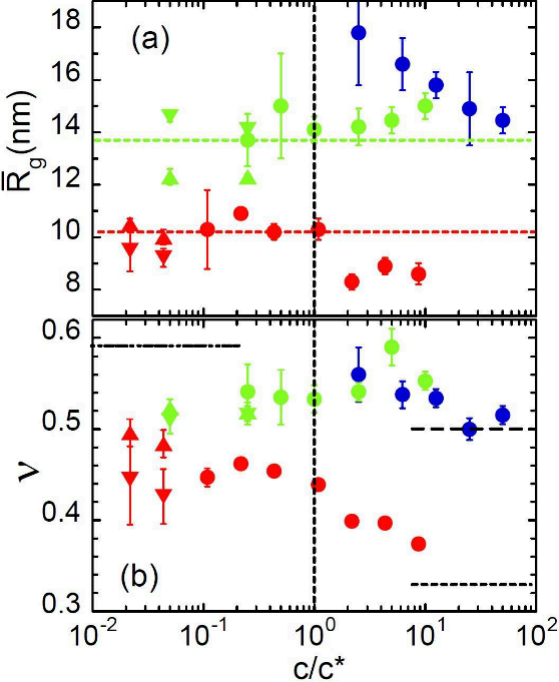
Parameters characterizing the macromolecular conformation in solutions of high-molecular weight precursor chains (blue symbols), MiNPs (red symbols) and CuNPs (green symbols) as a function of ratio between concentration and overlap concentration c*: (a) average radius of gyration ${\mathrm{R̅}}_{g}$ ; (b) scaling exponent ν. Results correspond to full-contrast (protonated polymer in deuterated solvent: up-triangles, inverse label: down-triangles) and ZAC condition (circles). Vertical line indicates the value *c* = c*. Dotted lines in (a) show the ${\mathrm{R̅}}_{g}$ -value in dilute conditions and lines in (b) the values of ν = 0.59 (dashed-dotted), 0.50 (dashed), and 0.33 (dotted).

In summary, we have exploited the unique opportunity offered by high-concentration labeling neutron scattering methods to elucidate the impact of the reversibility of bonds on the macromolecular conformation of SCNPs under self-crowding conditions. Through a detailed analysis of the isolated chain form factors, we have characterized conformations of reversibly bonding polymers, from the high dilution limit, where they form reversible SCNPs, to far above the overlap concentration and in the bulk state, where they form reversible networks. Unlike in the case of simple linear chains (which shrink and cross over from self-avoiding to random walk conformations), the size and scaling exponents of the reversibly bonding polymers are essentially unperturbed by crowding in the whole concentration range explored. Pushing the experimental conditions to their limits, concentrations as high as 10 times the overlap concentration could be reached for the solutions; taking into account also the results at bulk conditions, these conclusions maybe extended for the whole high-concentration range, despite the small unfortunate gap. These findings reflect the unusual negligibility of many-body effects in intermolecular interactions. This result is highly relevant for the validity of theoretical approaches based on fluids of ultrasoft particles to predict structural and phase behavior of systems based on the assembly of reversibly bonding polymers, particularly for the design of dual or interpenetrating networks, which can be challenged by spinodal decomposition, and this can be anticipated through the use of effective potentials. Moreover, we have confirmed the collapse to crumpled globular conformations of irreversible SCNPs under self-crowding conditions, which up to now had only been observed under crowding by linear chains. Considering that SCNPs can be viewed as simplified model systems of biological macromolecules, the insights here provided can also shed light on the interplay between crowding and intermolecular bonding in systems as relevant as e. g. intrinsically disordered proteins, contributing to the important question of crowding effects in biology.^38–45^ As an outlook, addressing the dynamics of bond exchange in these systems is a challenging question that shall be the subject of future work.

## Supplementary Material



## Notes

Sample synthesis and characterization; SANS experimental details, including description of experiments to determine matching conditions (PDF)
