# Supplementary material for: Bond-Reversibility Effects on Self-Crowding of Unimacromolecular Nano-Objects
Source: ACS Macro Lett. 2025 Sep 11;14(10):1389–95. doi: 10.1021/acsmacrolett.5c00512 (PMC12548348; doi:10.1021/acsmacrolett.5c00512)
Supplement: Supplementary file 1 [file mz5c00512_si_001.pdf]

Supporting Information

Bond-Reversibility Effects on Self-Crowding of Unimacromolecular Nano-Objects

Ainara Ruiz-Bardillo,[†] Isabel Asenjo-Sanz,[†] Ester Verde-Sesto,[†,‡] Lionel Porcar,[¶] Joachim Kohlbrecher,[§] Jose A. Pomposo,[†,∥,‡] Angel J. Moreno,[†] Arantxa Arbe[†]* and Juan Colmenero[†,⊥]

[†]Centro de Física de Materiales (CFM-MPC), CSIC-UPV/EHU, Paseo Manuel de Lardizabal 5, E-20018 San Sebastián, Spain

[‡]IKERBASQUE-Basque Foundation for Science, María Díaz de Haro 3, 48013 Bilbao, Spain

[¶]Institut Laue-Langevin, 71 avenue des Martyrs, Grenoble Cedex 9, 38042, France

[§]Laboratory for Neutron Scattering, Paul Scherrer Institut, CH-5232 Villigen, Switzerland

[∥]Departamento de Polímeros y Materiales Avanzados: Física, Química y Tecnología (UPV/EHU), Apartado 1072, E-20080 San Sebastián, Spain

[⊥]Donostia International Physics Center (DIPC), Paseo Manuel de Lardizabal 4, 20018 San Sebastián, Spain

* E-mail: a.arbe@ehu.eus

INDEX

1. Materials and Techniques for Sample Characterization S3

1.1. Materials...S3

1.2. Techniques for Sample Characterization S3

2. Synthetic Procedures...S4

2.1. Synthesis of Precursor Copolymers: Poly($MMA_{0.7}$-ran-$AEMA_{0.3}$) and Poly([MMA-$_{d8}$]$_{0.7}$-ran-$AEMA_{0.3}$)...........................S4

2.2. Synthesis of SCNPs..S5

2.3. Samples for SANS Experiments...S7

3. SANS Experiments...S7

4. References S10

1. Materials and Techniques for Sample Characterization

1.1. Materials

Deuterated methyl methacrylate-$_{d8}$ (MMA-$_{d8}$) (≥99 atom % D), methyl methacrylate (MMA) (99%), (2-acetoacetoxy)ethyl methacrylate (AEMA) (95%), 2,2-azobis(2-methylpropionitrile (AIBN) (purum, ≥98.0%), potassium hydroxide (KOH) (ACS reagent, ≥85%, pellets), cupric acetate ($Cu(OAc)_2$) (98%) and ethyl acetate (EtOAc) (ACS reagent, 99.5%), diethyl ether (Et_2O) (ACS reagent, >99.0%), 1,2,4,5-Tetrabromobenze (97%) were purchased from Merck (Aldrich) and used as received, unless otherwise specified. Deuterated AEMA was not available. Methanol (MeOH) (synthesis grade), tetrahydrofuran (THF) (HPLC grade), and hydrochloric acid (HCl) (37%, extra pure) were obtained from Scharlab. Trimethylolpropane triacrylate (TMT) (technical grade) and 2-cyanoprop-2-yl-dithiobenzoate (CPDB) (≥97%) were purchased from Alfa Aesar and Strem Chemicals, respectively. Dimethylformamide-$_{d7}$ (dDMF) (99.5% D) was purchased from Eurisotop and dimethylformamide (hDMF) (99.8% extra dry) was provided by Thermo Scientific. MMA and AEMA were purified by passing these monomers through basic alumina before use, and AIBN was recrystallized from MeOH.

1.2. Techniques for Sample Characterization

Size Exclusion Chromatography (SEC): SEC measurements were performed at 30 °C on an Agilent 1260 system equipped with PLgel 10 μm Guard and PLgel 10 μm MIXED-B LS columns, a differential refractive index (RI) detector (Optilab Rex, Wyatt), a multiangle laser light scattering (MALLS) detector (MiniDawn, Wyatt). Data analysis was performed with ASTRA Software (version 8.2.2) from Wyatt. THF was used as eluent at a flow rate of 1 mL/min using a dn/dc value of 0.083.

^{1}H Nuclear Magnetic Resonance (^{1}H NMR) Spectroscopy: ^{1}H NMR spectra were acquired at room temperature on a Bruker spectrometer operating at 400 MHz, using $CDCl_3$ as a solvent. To determine the composition of the deuterated copolymers, tetrabromobenzene was used as an internal standard.

2. Synthetic Procedures

2.1. Synthesis of Precursor Copolymers: Poly($MMA_{0.7}$-ran-$AEMA_{0.3}$) and Poly([MMA-$_{d8}$]$_{0.7}$-ran-$AEMA_{0.3}$)

For sample preparation, it was necessary to synthesize low and high molecular weight precursors (~50 and 250 kg/mol), both protonated and MMA-deuterated, with a final composition of approximately 70 mol% MMA or MMA-$_{d8}$ and 30 mol% AEMA. **Table S1** summarizes the codes of the synthesized precursor.

Table S1. Codes of the synthetized precursors in this study.

**hPre-50**	Low molecular weight protonated precursor
**dPre-50**	Low molecular weight deuterated precursor
**hPre-250**	High molecular weight protonated precursor
**dPre-250**	High molecular weight deuterated precursor

In a typical procedure, MMA or MMA-$_{d8}$, AEMA, CPDB and AIBN were dissolved in anhydrous EtOAc. The solution was degassed by bubbling nitrogen (N_2) for 15 min at room temperature (298 K). The copolymerization was conducted at 338 K for 18 h under a N_2 atmosphere and then quenched by rapid cooling in liquid N_2. The copolymer was isolated by precipitation three times into cold MeOH (200 mL), collected by filtration, and dried under dynamic vacuum at 303 K overnight.

Table S2 summarizes the quantities of reagents used in the synthesis of the precursor copolymers, the yields of the copolymerization reactions, and the weight average molecular weight (M_w) and dispersity ($Đ$) values of the obtained copolymers.

Table S2. Reagent quantities, reaction yield and SEC data for the synthesized precursor polymers.

Precursor	MMA/ MMA-$_{d8}$ (mmol)	AEMA (mmol)	CPDB (mmol)	AIBN (mmol)	EtOAc (mL)	AEMA (mol%)[a]	Yield (%)	M_w (kg/mol)[b]	$Đ$ [b]
hPre-50	27.9	9.4	9.3×10^{-2}	2.4×10^{-2}	6.0	28	80	52	1.1
dPre-50	24.0	7.8	5.6×10^{-2}	2.0×10^{-2}	4.0	28	85	54	1.1
hPre-250	18.7	5.0	1.7×10^{-2}	2.0×10^{-2}	6.3	30	86	237	1.3
dPre-250	17.3	5.8	1.1×10^{-2}	1.5×10^{-2}	6.3	30	85	250	1.4

[a] Calculated by NMR, [b] Determined by SEC

2.2. Synthesis of SCNPs

To ensure the formation of exclusively intramolecular crosslinks leading to unimolecular nano-objects, the syntheses of SCNPs from the precursor copolymers were carried out under high dilution conditions.

Reversible SCNPs (CuNPs) were synthesized by means of intrachain copper-complexation,[1,2] whereas irreversible SCNPs (MiNPs) were prepared with the use of a multifunctional cross-linker and the Michael addition reaction to produce permanent intrachain crosslinks[3] (see **Scheme S1**).

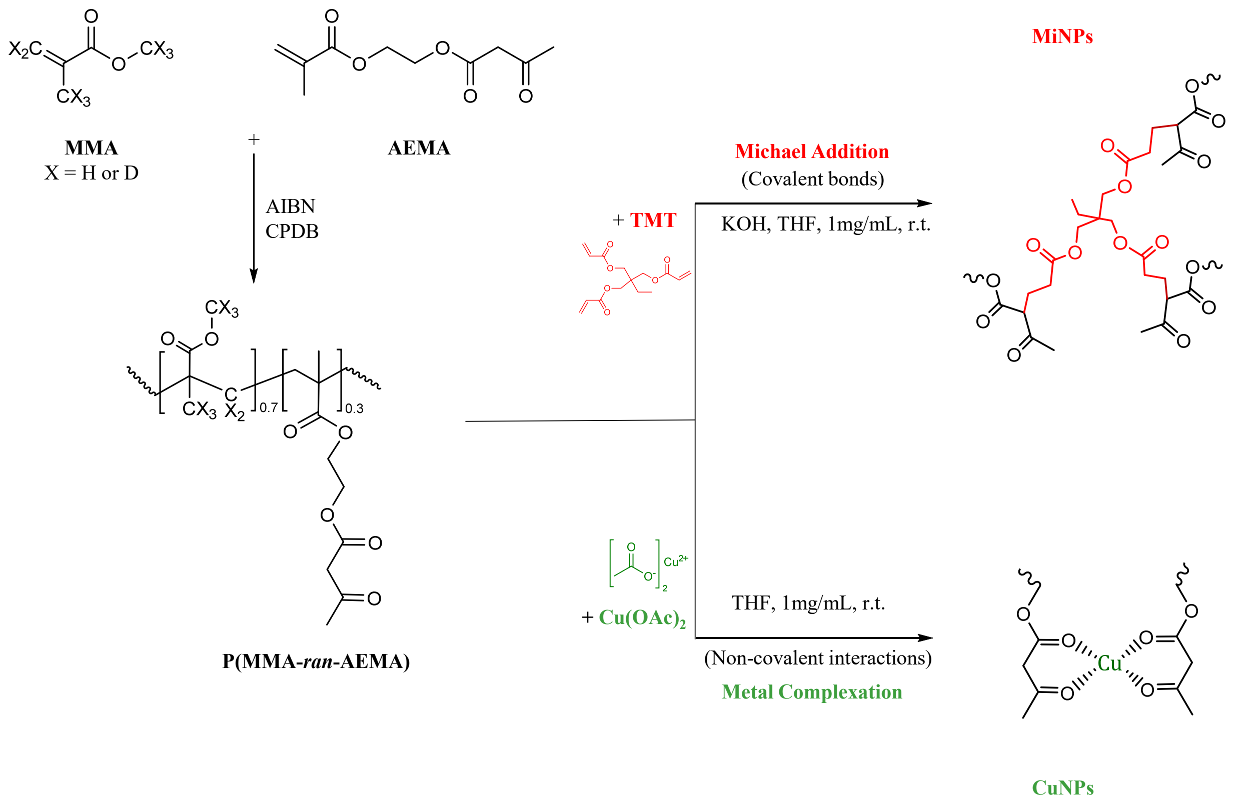

Scheme S1. Schematic illustration of the procedures followed to synthesize reversible (CuNPs) and irreversible (MiNPs) single-chain nanoparticles.

Synthesis of Reversible SCNPs (CuNPs): Reversible SCNPs were prepared by intrachain metal complexation following similar synthetic methods described in previous works.[1,2] 100 mg of the precursor were dissolved in THF (80 mL) followed by the slow addition of a solution of $Cu(OAc)_2$ (0.5 molar equivalents relative to the AEMA mol% content) in THF (20 mL), getting a final polymer concentration of 1 mg/mL. After the complete addition of the copper solution, the reaction mixture was stirred at room temperature for 24 hours. The resulting mixture was

then filtered and concentrated under vacuum to a final volume of 2–3 mL. The reversible CuNPs were isolated by precipitation in Et_2O, filtrated and subsequently dried under vacuum overnight at room temperature. The successful intramolecular collapse into SCNPs was confirmed by SEC (see **Figure S1**).

Synthesis of Irreversible SCNPs (MiNPs): Irreversible SCNPs were prepared by dissolving 100 mg of the precursor in THF (100 mL) at room temperature, achieving a final concentration of 1 mg/mL. KOH and TMT were added to the reaction mixture as catalyst and multifunctional cross-linker, respectively, in molar ratios of 0.5 and 0.33 relative to the AEMA content, to promote intramolecular cross-linking *via* a Michael addition reaction.[3] The reaction was allowed to proceed with stirring and it reached completion after 3 days, as monitored by size exclusion chromatography (SEC) (see **Figure S1**). Subsequently, a few drops of hydrochloric acid were added to deactivate the KOH catalyst. Finally, the mixture was filtered, concentrated, and the irreversible MiNPs were isolated by precipitation in Et_2O, followed by further drying under vacuum at room temperature.

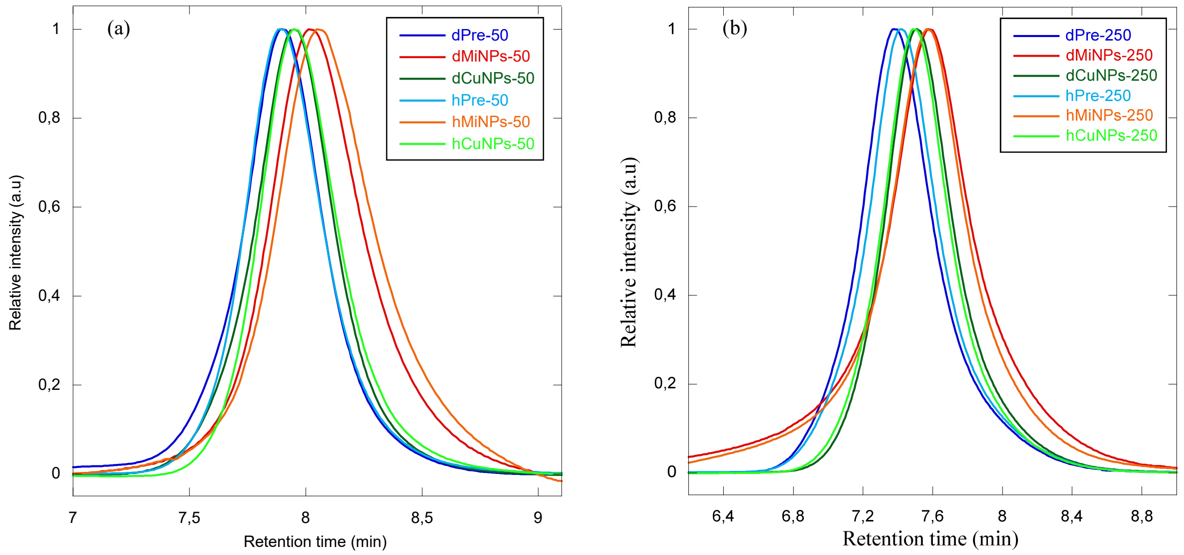

Figure S1. SEC chromatograms of: (a) Low-molecular-weight protonated and deuterated precursors and the corresponding CuSCNPs and MiSCNPs. (b) High-molecular-weight protonated and deuterated precursors and the corresponding CuSCNPs and MiSCNPs.

2.3. Samples for SANS Experiments

Bulk Samples: The samples (low-molecular weight) were prepared by dissolving the deuterated and protonated materials (60 mg of each) in a minimal volume of hDMF. Once fully dissolved, the solutions were cast into 10 × 10 × 1 mm molds and the solvent was evaporated overnight at 120 °C. The thicknesses of the areas exposed to the beam were of 0.70 mm (Pre), 0.36 mm (MiNP) and 0.40 mm (CuNP).

Solution Samples: All sample preparations were carried out inside a glovebox under a nitrogen atmosphere. The deuterated and protonated materials were dissolved in 4 mL vials and stirred overnight to ensure complete dissolution. Subsequently, the samples were transferred to 1 mm path-length Hellma quartz cuvettes, also under nitrogen atmosphere. These calculations accounted for both the densities of the solvent and the individual components. For ZAC conditions sample composition are 50/50 (v/v) deuterated/protonated materials. Solvent compositions were initially designed according to the estimated theoretical matching point and subsequently adjusted if necessary.

3. SANS Experiments

Small Angle Neutron Scattering (SANS) experiments were performed at room temperature on D22 at the Institut Laue-Langevin for the solutions of high-molecular weight macromolecules and bulk samples of nanoparticles and on SANS-I at the Paul Scherrer Institut for all the low-molecular weight samples. In both cases, a wavelength of λ=6Å was used. A two-collimations set up was used on D22, with 17 and 4 m; combining two sample-detector distances (17.6 and 5.6 m), the Q-range $0.002 \leq Q \leq 0.5$ Å^{-1} was covered. In SANS-I, 6 m collimation and two sample-detector distances of 6 and 2 m were used to explore the range $0.008 \leq Q \leq 0.5$ Å^{-1}. Solutions were filling Hellma cuvettes of 1 mm thickness.

The ZAC condition requires:

$$\rho_{p,D} - \rho_s = \rho_s - \rho_{p,H} \qquad \text{(S.1)}$$

where $\rho_{p,H}$ ($\rho_{p,D}$) refers to the scattering length density (SLD) of the protonated (deuterated) chains and ρ_s to the SLD of the solvent. In a dilute solution of polymer p (which can be deuterated of protonated) the coherently scattered intensity is given by

$$I(Q) = (\rho_p - \rho_s)^2 n V_p^2 P(Q) \quad \text{(S.2)}$$

where ρ_p is the SLD of the polymer; n is the number of chains per unit volume and V_p is the volume of a polymer chain. Thus, the $I(Q = 0)$-value for a solution of polymer p is $I(Q = 0) = (\rho_p - \rho_s)^2 n V_p^2$. Since deuterated and protonated chains have the same degree of polymerization, the matching condition (eq. S.1) is determined by the intersection of the curves $\sqrt{I(Q)}$ obtained on solutions of either protonated or deuterated chains in solvent of varying SLD with the same polymer concentration.

Dilute solutions of either deuterated or protonated SCNPs at the same concentration were measured in DMF solvents with varying SLD, i. e., with different volume fraction x of deuterated DMF in deuterated / protonated DMF mixtures. For the deuterated SCNPs, x-values of x = 0, 0.1, 0.2, 0.3, 0.4 and 0.5 were considered; for the protonated SCNPs, x = 0.5, 0.6, 0.7, 0.8, 0.9 and 1. **Figure S2** shows as an example the results obtained on the high-molecular weight MiNPs. The chosen concentration in this case was 10 mg/mL.

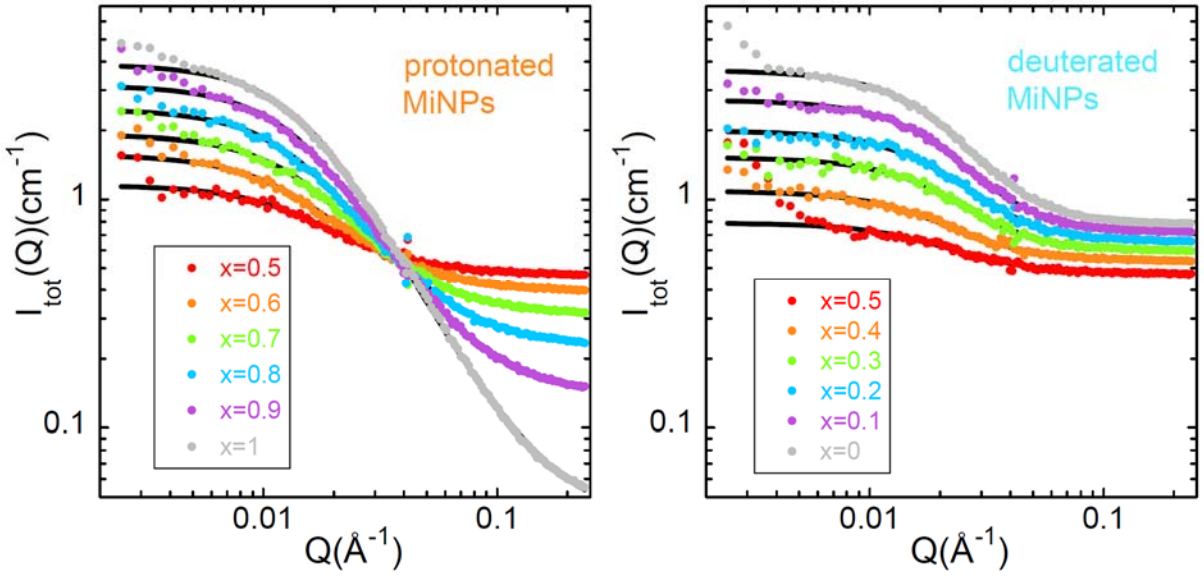

Figure S2. Total neutron intensity scattered by solutions of protonated chains (left) and deuterated chains (right) at 10 mg/mL of high-molecular weight MiNPs for the solvent compositions indicated (x: dDMF volume fraction of the solvent). Lines are fits of generalized Gaussian coils plus a background.

As can be seen in this figure, the total measured scattered intensity has a flat background that reflects the incoherent constant signal arising mainly from the protons in the samples. This background has to be subtracted from the measured intensity to obtain the coherent contribution

related to structural features. We also note that in general the uncertainties in the low Q results are relatively high. The small upturn shown by some of the data could reflect some aggregation of the macromolecules. This is most visible in the x=0.5 results of the deuterated SCNPs, where the signal is very weak and associated uncertainties are larger. The effect is, in any case, small and the aggregation, if any, should be very weak. However, this upturn prevents a direct accurate determination of the $I(Q$=0)-value from the lowest Q measured results in some of the cases. Therefore, for the $I(Q = 0)$-value determination, the total scattered intensities were fitted to

$$I_{tot}(Q) = I(Q = 0)P(Q) + BG \quad \text{(S.3)}$$

where BG accounts for the incoherent contribution. For the form factor $P(Q)$ a generalized Gaussian coil function[4] was assumed:

$$P(Q) = \frac{1}{\upsilon U^{\frac{1}{2\upsilon}}}\gamma\left(\frac{1}{2\upsilon}, U\right) - \frac{1}{\upsilon U^{\frac{1}{\upsilon}}}\gamma\left(\frac{1}{\upsilon}, U\right) \quad \text{(S.4)}$$

where

$$U = \frac{(2\upsilon+1)(2\upsilon+2)}{6}Q^2\bar{R}_g^2 \quad \text{(S.5)}$$

$$\gamma(a, x) = \int_0^x t^{a-1}e^{-t}dt \quad \text{(S.6)}$$

From the such obtained $I(Q = 0)$-values, the plot in **Figure S3** was obtained, allowing the determination of the matching condition for the sample (x_m=0.43). Analogous experiments gave very close values for the other SCNPs: x_m=0.45 and 0.43 for the high- and low-molecular weight CuNPs, respectively and x_m=0.42 for the low-molecular weight MiNPs. The slightly lower value of x_m for the MiNPs shall reflect the additional protons included in the crosslinking moiety.

Once the ZAC condition was determined, SCNPs and precursor solutions with ϕ=0.5 and polymer concentration c = 20, 50, 100, 200 and 400 mg/mL were investigated. As commented in the text, handling the 400 mg/mL-sample was not possible for the CuNPs. For the high-molecular weight SCNPs samples, 5 and 10 mg/mL were also studied in ZAC condition.

In addition, to explore dilute conditions full-contrast experiments were performed for the low-molecular weight samples at 5 mg/mL, at 1 mg/mL for the high-molecular weight CuNPs and at 1 and 2 mg/mL for the high-molecular weight MiNPs.

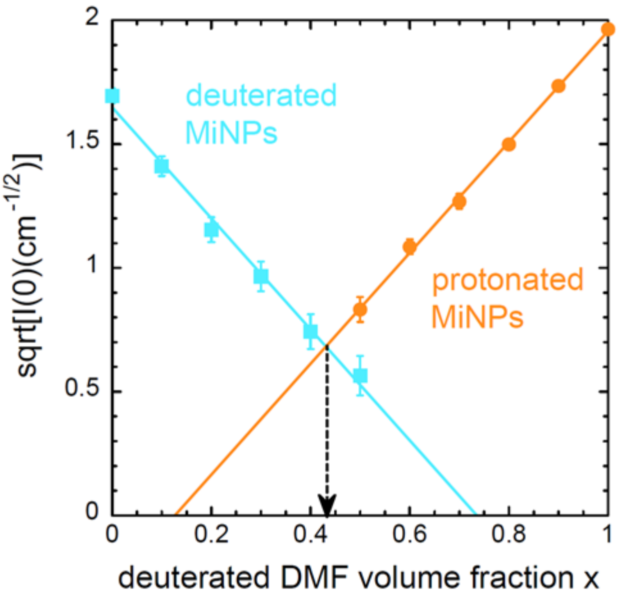

Figure S3. Solvent composition dependence of the square root of the Q=0-value of the coherent intensity of deuterated and protonated high-molecular weight MiNPs. Solid lines are linear regression fits. Vertical arrow marks the matching condition (x_m=0.43).

4. References

(1) Sanchez-Sanchez, A.; Arbe, A.; Colmenero, J.; Pomposo, J. A. Metallo-Folded Single-Chain Nanoparticles with Catalytic Selectivity. *ACS Macro Lett.* **2014**, *3*, 439-443.

(2) Pomposo, J. A.; Rubio-Cervilla, J.; Moreno, A. J.; Lo Verso, F.; Bacova, P.; Arbe, A.; Colmenero, J. Folding Single Chains to Single-Chain Nanoparticles via Reversible Interactions: What Size Reduction Can One Expect? *Macromolecules* **2017**, *50*, 1732-1739.

(3) Sanchez-Sanchez, A.; Akbari, S.; Etxeberria, A.; Arbe, A.; Gasser, U.; Moreno, A. J.; Colmenero, J.; Pomposo, J. A. "Michael" Nanocarriers Mimicking Transient-Binding Disordered Proteins. *ACS Macro Lett.* **2013**, *2*, 491-495.

(4) Hammouda, B. Small-angle scattering from branched polymers, *Macromolecular Theory and Simulations* **2012**, *21*, 372–381.
